# Supplementary material for: Antifungal and Antibiofilm Activity of Colombian Essential Oils against Different Candida Strains
Source: Antibiotics (Basel). 2023 Mar 29;12(4):668. doi: 10.3390/antibiotics12040668 (PMC10135359; doi:10.3390/antibiotics12040668)
Supplement: Supplementary file 1 [file antibiotics-12-00668-s001.zip › antibiotics-2272356-supplementary.pdf]

Supplementary data

Table S1. List of Colombian EOs assessed in this study. Major chemical compounds identified, and its relative amount reported as a percentage (%).

Plant Code	Plant species	Voucher number	Major EO compounds.
SA	*Steiractinia aspera*	UIS Herbarium 20891	α-Pinene (24.9%), β-pinene (14.8%), germacrene D (13.1%), β-phellandrene (10.1%), α-phellandrene (6.3%), sabinene (4.6%), *p*-cymene (4.5%), *trans*-β-caryophyllene (3.1%); α-copaene (2.6%), and limonene (2.4%).
TD-I	*Turnera diffusa*	UIS Herbarium 22037	Dehydrofukinone (25.4%), aristolochene (17.9%), valencene (7.4%), β-selinene (5.2%), *trans*-β-caryophyllene (4.0%), β-elemene (4.0%), premnaspirodiene (3.7%), guaiol (3.5%), germacra-4,5,10-trien-1-α-ol (3.5%), and caryophyllene oxide (3.3%).
LOP	*Lippia origanoides*, phellandrene chemotype	COL 560259	*trans*-β-Caryophyllene (18.6%), α-humulene (10.1%), α-phellandrene (9.3%), *p*-cymene (8.7%), 1,8-cineole (6.5%), limonene (4.4%), caryophyllene oxide (3.8%), β-phellandrene (3.1%), camphene (2.5%), and germacrene D (2.2%).
CM-I	*Calycolpus moritzianus*	UIS Herbarium 21982	1,8-Cineole (19.1%), limonene (17.6%), *trans*-β-caryophyllene (6.3%), viridiflorol (5.7%), α-pinene (5.1%), *trans,trans*-geranyl linalool (4.0%), *trans*-nerolidol (3.5%), α-copaene (3.2%), selina-3,7(11)-diene (2.8%), and viridiflorine (2.7%).
PA	*Piper aduncum*	COL 587136	Piperitone (14.8%), *trans*-β-caryophyllene (7.4%), viridiflorol (6.5%), limonene (6.0%), δ-cadinene (5.5%), α-pinene (4.6%), α-phellandrene (4.4%), caryophyllene oxide (3.8%), 1,8-cineole (3.6%), and *p*-cymene (3.0%).
EQ	*Elaphandra quinquenervis*	COL 587094	Germacrene D (20.7%), α-phellandrene (9.1%), α-pinene (6.8%), *trans*-β-caryophyllene (5.1%), Δ^3-carene (4.9%), limonene (4.5%), β-cubebene (3.5%), α-humulene (2.6%), premnaspirodiene (2.6%), and δ-cadinene (2.6%).
HD	*Hyptis dilatata*	COL 582530	*trans*-β-Caryophyllene (20.2%), camphor (16.1%), Δ^3-carene (15.5%), α-pinene (10.5%), palustrol (8.7%), α-gurjunene (4.7%), ledol (3.4%), limonene (2.4%), camphene (1.7%), viridiflorine (1.5%), and aromadendrene (1.5%).
LOC	*Lippia origanoides*, carvacrol chemotype	UIS Herbarium 22034	Carvacrol (35%), *p*-cymene (14.4%), thymol (8.0%), γ-terpinene (5.3%), *trans*-β-caryophyllene (4.4%), β-myrcene (2.4%), carvacryl acetate (2.0%), thymyl methyl ether (1.9%), and α-terpinene (1.7%).
LOCpT	*Lippia origanoides*, β-Caryophyllene + thymol chemotype	UIS Herbarium 22035	*trans*-β-Caryophyllene (15.1%), thymol (14%), 1,8-cineole (13%), *p*-cymene (12.6%), α-humulene (8.1%), α-phellandrene (7.1%), α-eudesmol (2.6%), caryophyllene oxide (2.5%), γ-terpinene (2.4%), and limonene (2.1%).
LOT-I	*Lippia origanoides*, thymol chemotype	COL 587107	Thymol (75.3%), *trans*-β-caryophyllene (5.4%), carvacrol (4.9%), α-humulene (3.2%), *p*-cymene (2.3%), thymyl acetate

Plant Code	Plant species	Voucher number	Major EO compounds.
			(1.6%), thymyl methyl ether (1.3%), caryophyllene oxide (1.3%), and *trans*-β-bergamotene (1.0%).
TD-II	*Turnera diffusa*	UIS Herbarium 22032	Aristolechene (20.9%), dehydrofukinone (19.3%), valencene (6.5%), β-selinene (5.8%), β-elemene (5.0%), *trans*-β-caryophyllene (4.9%), premnaspirodiene (4.7%), *p*-cymene (3.6%), germacra-4,5,10-trien-1-α-ol (3.6%), and guaiol (3.3%).
SV	*Satureja viminea*	COL 566449	*p*-Menth-3-en-8-ol (32.4%), pulegone (16.1%), *trans*-9-*epi*-caryophyllene (8.9%), *trans*-β-caryophyllene (8.4%), caryophyllene oxide (4.3%), spathulenol (3.6%), benzyl benzoate (2.4%), δ-cadinene (2.2%), *trans*-pulegol (1.8%), and *p*-Mentha-3,8-diene (1.5%).
PS	*Psidium sartorianum*	COL 578359	*trans*-β-Caryophyllene (12.7%), caryophyllene oxide (12.0%), dehydrofukinone (7.5%), caryophylla-4(12),8(13)-dien-5-β-ol (4.8%), germacrene B (4.1%), 1,8-cineole (3.7%), *p*-cymene (2.9%), β-pinene (2.7%), Selina-3,7(11)-diene (2.5%), β-selinene (2.1%), and premnaspirodiene (2.0%).
VC	*Varronia curassavica*	COL 559446	*trans*-β-Caryophyllene (19.2%), germacrene D (12.3%), *trans*-β-guaiene (11.8%), α-pinene (9.4%), α-copaene (7.0%), β-pinene (4.1%), bicyclogermacrene (3.9%), β-elemene (2.8%), δ-cadinene (2.8%), and α-humulene (2.7%).
OB	*Ocimum basilicum*	UIS Herbarium 22227	Linalool (42.7%), estragole (18.6%), 1,8-cineole (8.1%), germacrene D (4.9%), *epi*-α-cadinol (4.2%), γ-cadinene (3.7%), α-humulene (2.5%), β-elemene (2.2%), bicyclogermacrene (2.2%), and *trans*-α-bergamotene (1.1%).
CM-II	*Calycolpus moritzianus*	UIS Herbarium 21982	1,8-Cineole (15.4%), limonene (14.7%), viridiflorol (7.1%), *trans-trans*-Geranyllinalool (6.7%), *trans*-β-caryophyllene (6.2%), β-selinene (5.8%), *trans*-nerolidol (4.0%), α-pinene (3.5%), Selina-3,7(11)-diene (3.0%), and α-Copaene (3.0%).
TD-III	*Turnera diffusa*	Herbarium UIS 22037	Aristolochene (20.6%), dehydrofukinone (17.3%), *p*-cymene (5.8%), β-selinene (5.6%), valencene (5.2%), premnaspirodiene (4.2%), caryophyllene oxide (3.6%), *trans*-β-caryophyllene (2.8%), germacra-4,5,10-trien-1-α-ol (2.4%), and α-selinene.
LOTC	*Lippia origanoides*, thymol + *p*-cymene chemotype	Herbarium UIS 22039	Thymol (49.4%), *p*-cymene (19.1%), γ-terpinene (9.2%), β-myrcene (5.2%), α-terpinene (2.9%), carvacrol (2.7%), thymyl methyl ether (1.8%), *trans*-β-caryophyllene (1.6%), *cis*-β-ocimene (1.2%), and limonene (0.9%).
LOT-II	*Lippia origanoides*, thymol chemotype	Herbarium UIS 22036	Thymol (71.7%), *p*-cymene (10.5%), carvacrol (4.4%), β-myrcene (2.1%), γ-terpinene (2.0%), caryophyllene oxide (1.6%), thymyl methyl ether (0.9%), *trans*-β-caryophyllene (0.9%), humulene epoxide II (0.7%), and terpinen-4-ol (0.7%).
LM	*Lippia micromera*	COL 560986	*p*-Cymene (26.8%), thymyl methyl ether (26.3%), thymol (17.8%), thymyl acetate (5.7%), γ-terpinene (5.4%), 1,8-cineole (5.1%), α-terpinene (2.0%), β-myrcene (2.0%), *trans*-β-

Plant Code	Plant species	Voucher number	Major EO compounds.
			caryophyllene (1.7%), α-thujene (1.3%), and caryophyllene oxide (0.9%).
PMa	*Piper marginatum*	UIS Herbarium 21966	Isospathulenol (8.7%), α-pinene (5.8%), *trans*-β-caryophyllene (5.6%), limonene (5.6%), β-pinene (4.9%), germacrene D (4.6%), bicyclogermacrene (4.2%), δ-elemene (3.9%), and α-phellandrene (3.6%).
PR	*Piper reticulatum*	UIS Herbarium 21969	Germacrene D (14.5%), β-eudesmol (9.2%), β-elemene (7.4%), *trans*-β-caryophyllene (7.4%), germacrene B (4.9%), *trans*-nerolidol (4.9%), linalool (4.8%), β-selinene (2.9%), bicyclogermacrene (2.5%), and ishwarane (2.1%).
CaM	*Cantinoa mutabilis*	Herbarium UIS 21971	*trans*-β-Caryophyllene (21.5%), germacrene D (20.9%), α-pinene (7.0%), bicyclogermacrene (6.9%), α-copaene (6.5%), β-cubebene (5.3%), caryophyllene oxide (4.8%), δ-cadinene (4.0%), *epi*-α-acoradiene (2.6%), germacrene B (2.4%), and α-humulene (2.2%).
AP-I	*Ageratina popayanensis* (Hieron.) R. King & H. Rob.	Herbarium UIS 21975	α-Pinene (27.0%), camphene (11.4%), α-phellandrene (10.5%), β-pinene (8.4%), limonene (7.0%), *p*-cymene (4.7%), *trans*-verbenol (4.0%), *trans*-β-caryophyllene (3.1%), β-myrcene (2.2%), and verbenone (2.0%).
SF-I	*Simsia fruticulosa* (Spreng.) S. F. Blake	UIS Herbarium 21981	α-Thujene (24.6%), α-pinene (21.7%), germacrene D (4.5%), *trans*-thujanol (3.9%), β-myrcene (3.7%), sabinene (3.6%), *p*-cymene (2.6%), caryophyllene oxide (2.5%), and *trans*-sabinol (2.4%).
Vcu	*Varronia curassavica* Jacq.	UIS Herbarium 20892	*trans*-β-Caryophyllene (17.1%), α-pinene (11.0%), germacrene D (10.0%), α-copaene (8.8%), β-pinene (6.5%), caryophyllene oxide (4.5%), α-humulene (3.6%), β-elemene (3.2%), α-bisabolol (2.9%), and δ-cadinene (2.8%).
OC	*Ocimum campechianum*	UIS Herbarium 20889	Eugenol (35.3%), 1,8-cineole (15.6%), β-selinene (11.0%), *trans*-β-caryophyllene (7.4%), germacrene D (5.6%), α-selinene (4.8%), β-pinene (2.4%), β-elemene (1.9%), and α-humulene (1.5%).
LACL	*Lippia alba*, carvone + limonene chemotype	UIS Herbarium 22031	Limonene (40.1%), carvone (37.7%), germacrene D (8.1%), β-bourbonene (3.0%), piperitone (1.9%), β-myrcene (0.9%), piperitenone (0.8%), linalool (0.7%), borneol (0.7%), and *trans*-β-farnesene (0.7%).
LACi	*Lippia alba*, citral chemotype	UIS Herbarium 22032	Geranial (24.5%), geraniol (19.0%), neral (11.9%), *trans*-β-caryophyllene (9.1%), germacrene D (4.3%), geranyl acetate (2.8%), α-humulene (2.8%), β-elemene (2.6%), nerol (2.5%), and limonene (2.4%).
PC	*Pogostemon cablin*	UIS Herbarium 20890	Patchoulol (35.4%), α-bulnesene (15.9%), α-guaiene (13.5%), seychellene (8.5%), α-patchoulene (6.3%), pogostol (3.2%), aciphyllene (2.9%), *trans*-β-caryophyllene (2.6%), β-patchoulene (1.7%), and γ-patchoulene (1.3%).
HS	*Hyptis suaveolens*	COL 560241	*trans*-β-Caryophyllene (11.1%), 1,8-cineole (10,0%) fenchone (9.8%), limonene (8.5%), bicyclogermacrene (6.0%), sabinene

Plant Code	Plant species	Voucher number	Major EO compounds.
			(5.3%), β-pinene (5.1%), α-copaene (4.4%), germacrene D (3.4%), and α-pinene (2.9%).
MM-I	*Minthostachys mollis* (Benth.) Griseb.	UIS Herbarium 22041	*trans*-Piperitone oxide (49.6%), menthone (8.9%), piperitenone oxide (4.8%), *trans*-β-caryophyllene (4.0%), limonene (3.3%), thymol (2.3%), 6-hydroxycarvotanacetone (2.3%), germacrene D (2.1%), β-pinene (2.0%), linalool (1.9%), and pulegone (1.7%).
MM-II	*Minthostachys mollis* (Benth.) Griseb.	UIS Herbarium 22042	Menthone (46.1%), pulegone (13.3%), piperitone (12.1%), *trans*-β-caryophyllene (7.0%), germacrene D (3.8%), iso-menthone (3.5%), bicyclogermacrene (3.4%), α-humulene (1.9%), α-pinene (1.3%), and β-pinene (1.2%).
HC	*Hypericum* cf. *carinosum* L. R. Keller	UIS Herbarium 22045	α-Pinene (28.1%), tricyclene (16.4%), limonene (10.4%), β-pinene (9.5%), *trans*-β-caryophyllene (6.2%), camphene (4.2%), nonane (3.4%), β-myrcene (2.9%), α-humulene (2.9%), and *p*-cymene (2.5%).
SO	*Stevia ovata* Willd.	UIS Herbarium 22047	Germacrene D (26.4%), *trans*-β-caryophyllene (21.2%), *trans*-nerolidol (11.5%), bicyclogermacrene (6.3%), α-humulene (4.1%), guaiol (3.7%), caryophyllene oxide (2.3%), β-elemene (2.0%), β-bourbonene (1.9%), and δ-cadinene (1.8%).
AP-II	*Ageratina popayanensis* (Hieron.) R. King & H. Rob.	UIS Herbarium 22052	α-Pinene (20.5%), camphene (15.4%), limonene (11.3%), α-phellandrene (10.5%), β-pinene (10.0%), *p*-cymene (7.9%), *trans*-verbenol (3.3%), β-myrcene (1.9%), sabinene (1.6%), and myrtenol (1.6%).
LAC	*Lippia alba*, carvone chemotype	COL 582597	Limonene (33.8%), carvone (33.2%), germacrene D (15.7%), β-burbonene (2.7%), piperitenone (1.8%), β-elemene (1.8%), piperitone (1.4%), *trans*-β-farnesene (1.1%), bicyclogermacrene (0.9%), and *trans*-9-*epi*-caryophyllene (0.8%).
PAu	*Piper auritum*	COL 512209	Safrole (52.3%), terpinolene (10.7%), γ-terpinene (7.2%), α-phellandrene (3.6%), α-terpinene (3.1%), β-myrcene (3.0%), limonene (2.2%), α-pinene (2.1%), *trans*-β-caryophyllene (1.5%), and *p*-cymene (1.0%).
SF-II	*Simsia fruticulosa* (Spreng.) S. F. Blake	UIS Herbarium 21981	Germacrene D (17.7%), α-thujene (15.8%), α-pinene (9.0%), β-myrcene (7.4%), β-phellandrene (4.4%), sabinene (4.0%), *trans*-β-caryophyllene (3.8%), bicyclogermacrene (3.0%), and *trans*-nerolidol (2.1%).
OA	*Ocimum americanum* Jacq.	UIS Herbarium 22070	Eugenol (36.9%), *trans*-β-caryophyllene (28.8%), β-elemene (14.2%), caryophyllene oxide (3.9%), α-humulene (2.8%), borneol (10.5%), elemol (1.1%), α-eudesmol (0.7%), and α-selinene (0.6%).
PS	*Piper sphaeroides* C. DC.	UIS Herbarium 22074	Linalool (12.8%), *trans*-β-caryophyllene (11.8%), β-pinene (4.8%), δ-cadinol (4.7%), *trans*-nerolidol (4.6%), dillapiole (4.2%), α-copaene (3.3%), caryophyllene oxide (3.1%), and α-terpinene (2.7%).

Plant Code	Plant species	Voucher number	Major EO compounds.
PD	*Piper dilatatifolium* Trel. & Yunck	UIS Herbarium 22075	α-Pinene (17.4%), *trans*-β-caryophyllene (15.5%), germacrene D (11.3%), β-elemene (5.6%), carotol (5.3%), bicyclogermacrene (4.6%), δ-cadinene (4.3%), α-humulene (2.9%), germacrene B (2.4%), α-Copaene (2.2%), and β-bourbonene (2.1%).
VCy	*Varronia* aff. *cylindrostachya* Ruiz & Pav.	UIS Herbarium 22078	α-Pinene (19.0%), *trans*-β-caryophyllene (17.1%), germacrene D (11.1%), di-*epi*-1,10-cubenol (6.7%), β-elemene (5.9%), δ-cadinene (5.1%), bicyclogermacrene (4.8%), β-phellandrene (3.7%), α-humulene (3.4%), and germacrene B (2.4%).
AdP	*Adenophyllum porophyllum* (Cav.) Hemsl.	UIS Herbarium 22080	Pinocarvone (47.1%), limonene (19.0%), *p*-cymene (5.4%), sabinene (5.2%), *cis*-3-pinocamphone (5.0%), α-phellandrene (3.5%), β-pinene (3.3%), 1,8-cineole (2.5%), *p*-cumenol (1.0%), and α-campholenal (0.8%).
LC	*Lantana* aff. *colombiana* López-Pal.	UIS Herbarium 22082	*trans*-β-Caryophyllene (26.5%), germacrene D (13.8%), α-humulene (10.9%), β-elemene (6.1%), caryophyllene oxide (5.4%), limonene (3.6%), bicyclogermacrene (3.1%), sabinene (2.4%), β-bourbonene (2.1%), *trans*-β-bisabolene (1.9%), and α-copaene (1.8%).
DC	*Dalea carthagenensis*	UIS Herbarium 22083	β-Pinene (28.3%), β-myrcene (24.9%), *trans*-β-caryophyllene (9.6%), limonene (6.4%), germacrene B (4.3%), α-pinene (3.6%), *trans*-β-ocimene (1.9%), germacrene D (1.7%), *epi*-α-cadinol (1.6%), γ-elemene (1.6%), and benzyl benzoate (1.6%).
PP	*Piper piedecuestanum*	UIS Herbarium 22086	Dillapiole (46.7%), elemicin (12.6%), eudesma-4(15),7-dien-1β-ol (6.9%), bicyclogermacrene (5.5%), spathulenol (4.8%), β-pinene (4.8%), α-pinene (1.8%), γ-muurolene (1.6%), and *trans*-β-caryophyllene (1.3%).
DF	*Dalea foliolosa*	UIS Herbarium 22090	Elemicin (57.9%), β-phellandrene (10.1%), *trans*-β-caryophyllene (8.8%), germacrene D (5.9%), bicyclogermacrene (4.1%), methyl-eugenol (1.7%), linalool (1.4%), terpinolene (1.4%), and α-humulene (0.8%).
MR	*Myrcianthes rhopaloides*	UIS Herbarium 22091	Limonene (17.6%), *cis-trans*-farnesol (12.6%), germacrene D (12.6%), β-myrcene (5.1%), α-phellandrene (4.8%), bicyclogermacrene (3.7%), α-humulene (3.6%), α-cadinol (3.3%), β-elemene (3.2%), and δ-cadinene (3.1%).
PAm	*Piper amalago*	UIS Herbarium 22093	Ishwarone (15.0%), α-pinene (12.4%), ishwarane(11.9%), ishwarol B (7.6%), Elemol (5.6%), linalool (4.6%), β-pinene (4.2%), *trans*-β-caryophyllene (3.8%), β-elemene (3.4%), limonene (3.2%), Valencene (2.4%), and β-phellandrene(2.4%).

UIS: Industrial University of Santander (Bucaramanga, Colombia)

Table S2. Bioactive and non-bioactive Colombian EOs major chemical compounds identified. Data is organized by bioactive properties.

Chemical composition of Eos — Bioactive Eos

Compound \ EOs	EQ	LM	OC	LACi	MM-I	HC	LOC	LOCpT	LOT-I	LOTC	LOT-II	LACL	TD-I	CM-II	TD-III	MM-II
	Planktonic activity						Planktonic and antibiofilm activity						Antibiofilm activity			
trans-β- Caryophyllene	5.1	1.7	7.4	9.1	4.0	6.2	4.4	15.1	5.4	1.6	0.9		4.0	6.2	2.8	7.0
Germacrene D	20.7		5.6	4.3	2.1							8.1				3.8
Limonene	4.5			2.4	3.3	10.4		2.1		0.9		40.1		14.7		
α-Pinene	6.8					28.1								3.5		1.3
α-Humulene	2.6		1.5	2.8		2.9		8.1	3.2							1.9
p-Cymene		26.8				2.5	14.4	12.6	2.3	19.1	10.5				5.8	
Caryophyllene oxide		0.9						2.5	1.3		1.6		3.3		3.6	
β-Pinene			2.4		2.0	9.5										1.3
Bicyclogermacrene																3.4
β-Elemene			1.9	2.6									4.0			
β-Myrcene		2.0				2.9	2.4			5.2	2.1	0.9				
α-Phellandrene	9.1							7.1								
δ-Cadinene	2.6															
1,8-Cineole		5.1	15.6					13.0						15.4		
α-Copaene														3.0		
Thymol		17.8			2.3		8.0	14.0	75.3	49.4	71.7					
Sabinene																
Linalool					1.9							0.7				
β-selinene			11.0										5.2	5.8	5.6	
γ-terpinene		5.4					5.3	2.4		9.2	2.0					
Germacrene B																
trans-Nerolidol														4.0		
β-Phellandrene																
Thymyl methyl ether		26.3					1.9		1.4	1.8	0.9					
Premnaspirodiene	2.6												3.7		4.2	
Camphene						4.2										

Chemical composition of Eos — Non-Active Eos

Compound \ EOs	SA	LOP	CM-I	PA	HD	TD-II	SV	PS	VC	OB	PMa	PR	CaM	AP-I	SF-I	Vcu	PC	HS	SO	AP-II	LAC	PAu	SF-II	OA	PS	PD	VCy	AdP	LC	DC	PP	DF	MR	PAm
trans-β- Caryophyllene	3.1	18.6	6.3	7.4	20.2	4.9	8.4	12.7	19.2		5.6	7.4	21.5	3.1		17.1	2.6	11.1	21.2			1.5	3.8	28.8	11.8	15.5	17.1		26.5	9.6	1.3	8.8		3.8
Germacrene D	13.1	2.2							12.3	4.9	4.6	14.5	20.9		4.5	10.0		3.4	26.4		15.7		17.7			11.3	11.1		13.8	1.7		5.9	12.6	
Limonene	2.4	4.4	17.6	6.0	2.4						5.6			7.0				8.5		11.3	33.8	2.2						19.0	3.6	6.4			17.6	3.2
α-Pinene	24.9		5.1	4.6	10.5				9.4		5.8		7.0	27.0	21.7	11.0		2.9		20.5		2.1	9.0			17.4	19.0			3.6	1.8			12.4
α-Humulene		10.1							2.7	2.5			2.2			3.6			4.1					2.8		2.9	3.4		10.9			0.8	3.6	
p-Cymene	4.5	8.7		3.0		3.6		2.9						4.7	2.6					7.9		1.0						5.4						
Caryophyllene oxide		3.8		3.8			4.3	12.0					4.8		2.5	4.5			2.3					3.9	3.1				5.4					
β-Pinene	14.8							2.7	4.1		4.9			8.4		6.5		5.1		10.0					4.8			3.3		28.3	1.8			4.2
Bicyclogermacrene									3.9	2.2	4.2	2.5	6.9					6.0	6.3		0.9		3.0			4.6	4.8		3.1		5.5	4.1	3.7	
β-Elemene						5.0			2.8	2.2		7.4				3.2			2.0		1.8			14.2		5.6	5.9		6.1				3.2	3.4
β-Myrcene														2.2	3.7					1.9		3.0	7.4							24.9			5.1	
α-Phellandrene	6.3	9.3		4.4							3.6			10.5						10.5		3.6						3.5					4.8	
δ-Cadinene				5.5			2.2		2.8		3.4		4.0			2.9			1.8							4.3	5.1						3.1	
1,8-Cineole		6.5	19.1	3.6				3.7		8.1								10.0										2.5						
α-Copaene	2.6		3.2						7.0				6.5			8.8		4.4							3.3	2.2			1.8					
Thymol																																		
Sabinene	4.6														3.6			5.3		1.3			4.0					5.2	2.4					
Linalool										42.7		4.8													12.8							1.4		4.6
β-selinene						5.8		2.1				2.9																						
γ-terpinene																						7.2												
Germacrene B								4.1				4.9	2.5													2.4	2.4			4.3				
trans-Nerolidol			3.5									4.9							11.5				2.1		4.6									
β-Phellandrene	10.1	3.1																					4.4				3.7					10.1		2.4
Thymyl methyl ether																																		
Premnaspirodiene						4.7		2.0																										
Camphene		2.5			1.7									11.4						15.4														

Compound / EOs	EQ	LM	OC	LACi	MM-I	HC	LOC	LOCpT	LOT-I	LOTC	LOT-II	LACL	TD-I	CM-II	TD-III	MM-II	SA	LOP	CM-I	PA	HD	TD-II	SV	PS	VC	OB	PMa	PR	CaM	AP-I	SF-I	Vcu	PC	HS	SO	AP-II	LAC	PAu	SF-II	OA	PS	PD	VCy	AdP	LC	DC	PP	DF	MR	PAm
	**Chemical composition of Eos**																																																	
	**Bioactive Eos**																**Non-Active Eos**																																	
	**Planktonic activity**						**Planktonic and antibiofilm activity**						**Antibiofilm activity**																																					
β-Burbonene												3.0																							1.9		2.7					2.1			2.1					
α-terpinene		2.0					1.7			2.9																												3.1			2.7									
Carvacrol							35.0		4.9	2.7	4.4																																							
Dehydrofukinone													25.4		17.3							19.3		7.5																										
Piperitone												1.9				12.1				14.8																	1.4													
Valencene													7.4		5.2							6.5																												2.4
Viridiflorol														7.1					5.7	6.5																														
Selina-3,7(11)-diene														3.0					2.8					2.5																										
Guaiol													3.5									3.3													3.7															
α-Thujene		1.3																													24.6								15.8											
Aristolochene													17.9		20.6							20.9																												
Germacra-4,5,10-trien-1-α-ol													3.5		2.4							3.6																												
Pulegone					1.7											13.3							16.1																											
α-Celinene			4.8												2.5																									0.6										
Δ[3]-Carene	4.9																				15.5																													
β-Cubebene	3.5																												5.3																					
Benzyl benzoate																							2.4																							1.6				
Dillapiole																																									4.2						46.7			
Elemicin																																															12.6	57.9		
Elemol																																								1.1										5.6
epi-α-cadinol																										4.2																				1.6				
Spathulenol																							3.6																								4.8			
Ishwarane																												2.1																						11.9
Terpinolene																																						10.7										1.4		
trans,trans-Geranyl linalool														6.7					4.0																															
trans-9-*epi*-caryophyllene																							8.9														0.8													
trans-Verbenol																														4.0						3.3														

Compound / EOs	Chemical composition of Eos																																																	
	Bioactive Eos																Non-Active Eos																																	
	Planktonic activity						Planktonic and antibiofilm activity						Antibiofilm activity																																					
	EQ	LM	OC	LACi	MM-I	HC	LOC	LOCpT	LOT-I	LOTC	LOT-II	LACL	TD-I	CM-II	TD-III	MM-II	SA	LOP	CM-I	PA	HD	TD-II	SV	PS	VC	OB	PMa	PR	CaM	AP-I	SF-I	Vcu	PC	HS	SO	AP-II	LAC	PAu	SF-II	OA	PS	PD	VCy	AdP	LC	DC	PP	DF	MR	PAm
Viridiflorine																			2.7		1.5																													
Borneol												0.7																												1.5										
Carvone												37.7																									33.2													
Eugenol			35.3																																					36.6										
Piperitenone												0.8																									1.8													
Trans-β-farnesene												0.7																									1.1													
α-Eudesmol								2.6																																0.7										
Thymyl acetate		5.7							1.6																																									
Menthone					8.9											46.1																																		
Humulene epoxide II											0.7																																							
Terpinen-4-ol											0.7																																							
Carotol																																										5.3								
Aciphyllene																																	2.9																	
Camphor																					16.1																													
Aromadendrene																					1.5																													
Caryophylla-4(12),8(13)-dien-5-β-ol																								4.8																										
cis-3-Pinocamphone																																												5.0						
cis-*trans*-Carnesol																																																	12.6	
di-*epi*-1,10-Cubenol																																											6.7							
epi-α-Acoradiene																													2.6																					
Estragole																										18.6																								
Eudesma-4(15),7-dien-1β-ol																																															6.9			
Fenchone																																		9.8																
Ishwarol B																																																		7.6
Ishwarone																																																		15.0
iso-Spathulenol																											8.7																							
Ledol																					3.4																													

EOs	Chemical composition of Eos															
	Bioactive Eos															
	Planktonic activity						Planktonic and antibiofilm activity						Antibiofilm activity			
Compound	EQ	LM	OC	LACi	MM-I	HC	LOC	LOCpT	LOT-I	LOTC	LOT-II	LACL	TD-I	CM-II	TD-III	MM-II
Methyl-eugenol																
Myrtenol																
Nonane						3.4										
palustrol																
Patchoulol																
p-cumenol																
Pinocarvone																
p-Menth-3-en-8-ol																
p-Mentha-3,8-diene																
Pogostol																
Safrole																
Seychellene																
trans-Pulegol																
trans-Sabinol																
trans-thujanol																
trans-α-Bergamotene																
trans-β-Bisabolene																
trans-β-Guaiene																
trans-β-Ocimene																
Tricyclene						16.4										
Verbenone																
α-Bisabolol																
α-Bulnesene																
α-Cadinol																
α-Campholenal																
α-Guaiene																
α-Gurjunene																
α-Patchoulene																

EOs	Non-Active Eos																																	
Compound	SA	LOP	CM-I	PA	HD	TD-II	SV	PS	VC	OB	PMa	PR	CaM	AP-I	SF-I	Vcu	PC	HS	SO	AP-II	LAC	PAu	SF-II	OA	PS	PD	VCy	AdP	LC	DC	PP	DF	MR	PAm
Methyl-eugenol																																1.7		
Myrtenol																				1.6														
Nonane																																		
palustrol					8.7																													
Patchoulol																	35.4																	
p-cumenol																												1.0						
Pinocarvone																												47.1						
p-Menth-3-en-8-ol							32.4																											
p-Mentha-3,8-diene							1.5																											
Pogostol																	3.2																	
Safrole																						52.3												
Seychellene																	8.5																	
trans-Pulegol							1.8																											
trans-Sabinol															2.4																			
trans-thujanol															3.9																			
trans-α-Bergamotene										1.1																								
trans-β-Bisabolene																													1.9					
trans-β-Guaiene									11.8																									
trans-β-Ocimene																														1.9				
Tricyclene																																		
Verbenone														2.0																				
α-Bisabolol																2.9																		
α-Bulnesene																	15.9																	
α-Cadinol																																	3.3	
α-Campholenal																												0.8						
α-Guaiene																	13.5																	
α-Gurjunene					4.7																													
α-Patchoulene																	6.3																	

Chemical composition of Eos

Bioactive Eos

Compound \ EOs	Planktonic activity						Planktonic and antibiofilm activity						Antibiofilm activity			
	EQ	LM	OC	LACi	MM-I	HC	LOC	LOCpT	LOT-I	LOTC	LOT-II	LACL	TD-I	CM-II	TD-III	MM-II
β-Eudesmol																
β-Patchoulene																
γ-Cadinene																
γ-Elemene																
γ-Muurolene																
γ-Patchoulene																
δ-Cadinol																
δ-Elemene																
6-Hydroxycarvotanacetone					2.3											
Carvacryl acetate							2.0									
Geranyl acetate				2.8												
cis-β-Ocimene										1.2						
Geranial				24.5												
Geraniol				19.0												
iso-Menthone																3.5
Neral				11.9												
Nerol				2.5												
Piperitenone oxide					4.8											
trans-Piperitone oxide					49.6											
trans-*β*-bergamotene									1.0							

Non-Active Eos

Compound \ EOs	SA	LOP	CM-I	PA	HD	TD-II	SV	PS	VC	OB	PMa	PR	CaM	AP-I	SF-I	Vcu	PC	HS	SO	AP-II	LAC	PAu	SF-II	OA	PS	PD	VCy	AdP	LC	DC	PP	DF	MR	PAm
β-Eudesmol												9.2																						
β-Patchoulene																	1.7																	
γ-Cadinene										3.7																								
γ-Elemene																														1.6				
γ-Muurolene																															1.6			
γ-Patchoulene																	1.3																	
δ-Cadinol																									4.7									
δ-Elemene											3.9																							
6-Hydroxycarvotanacetone																																		
Carvacryl acetate																																		
Geranyl acetate																																		
cis-β-Ocimene																																		
Geranial																																		
Geraniol																																		
iso-Menthone																																		
Neral																																		
Nerol																																		
Piperitenone oxide																																		
trans-Piperitone oxide																																		
trans-*β*-bergamotene																																		

Table S3. MIC50 and MFC values for the EOs studied against *C. albicans* ATCC 10231, *C. parapsilosis* ATCC 22019, and *C. auris* CDC B11903.

EO	*C. albicans* ATCC 10231		*C. parapsilosis* ATCC 22019		*C. auris* CDC B11903	
	MIC50 (µg/mL)	MFC (µg/mL)	MIC50 (µg/mL)	MFC (µg/mL)	MIC50 (µg/mL)	MFC (µg/mL)
SA	NA	NA	750	NA	750	NA
TD-I	750	NA	NA	NA	NA	NA
LOP	750	NA	750	NA	NA	NA
CM-I	NA	NA	NA	NA	NA	NA
PA	NA	NA	750	NA	NA	NA
EQ	NA	NA	375	NA	NA	NA
HD	NA	NA	NA	NA	NA	NA
LOC	375	NA	281	NA	281	NA
LOCpT	750	563	188	NA	750	NA
LOT-I	281	750	188	750	188	563
TD-II	NA	NA	750	NA	750	NA
SV	NA	NA	NA	NA	NA	NA
PS	NA	NA	NA	NA	NA	NA
VC	NA	NA	NA	NA	NA	NA
OB	NA	NA	NA	NA	NA	NA
CM-II	NA	NA	NA	NA	NA	NA
TD-III	750	NA	750	NA	NA	NA
LOTC	188	NA	281	NA	188	375
LOT-II	188	563	141	563	141	375
LM	750	NA	188	NA	750	NA
PM	NA	NA	NA	NA	NA	NA
PR	NA	NA	NA	NA	NA	NA
CaM	NA	NA	NA	NA	NA	NA
AP-I	750	NA	NA	NA	NA	NA
SF-I	NA	NA	NA	NA	NA	NA
Vcu	750	NA	NA	NA	NA	NA
OC	563	NA	563	NA	563	NA
LACL	750	NA	750	NA	188	563
LACi	750	NA	750	NA	563	NA
PC	750	NA	NA	NA	NA	NA
HS	NA	NA	NA	NA	NA	NA
MM-I	750	NA	375	750	375	NA
MM-II	NA	NA	750	NA	750	NA
HC	563	NA	375	750	375	NA
SO	NA	NA	750	NA	750	NA
AP-II	NA	NA	NA	NA	NA	NA
LAC	NA	NA	NA	NA	750	NA
PAu	NA	NA	NA	NA	750	NA
SF-II	NA	NA	NA	NA	NA	NA

EO	*C. albicans* ATCC 10231		*C. parapsilosis* ATCC 22019		*C. auris* CDC B11903	
	**MIC_{50} (μg/mL)**	**MFC (μg/mL)**	**MIC_{50} (μg/mL)**	**MFC (μg/mL)**	**MIC_{50} (μg/mL)**	**MFC (μg/mL)**
OA	NA	NA	750	NA	750	NA
PS	NA	NA	750	NA	NA	NA
PD	NA	NA	750	NA	NA	NA
VCy	NA	NA	NA	NA	NA	NA
AdP	NA	NA	NA	NA	750	NA
LC	NA	NA	750	NA	NA	NA
DC	NA	NA	NA	NA	NA	NA
PP	NA	NA	NA	NA	NA	NA
DF	750	NA	750	NA	750	NA
MR	NA	NA	NA	NA	NA	NA
PAm	NA	NA	NA	NA	NA	NA

NA: Non-Active. MIC_{50} NA μg/mL

Table S4. $MBIC_{50}$ values for the EOs studied against *C. albicans* ATCC 10231, *C. parapsilosis* ATCC 22019, and *C. auris* CDC B11903.

Essential Oil	*C. albicans* ATCC 10231	*C. parapsilosis* ATCC 22019	*C. auris CDC B11903*
	$MBIC_{50}$ (μg/mL)	$MBIC_{50}$ (μg/mL)	$MBIC_{50}$ (μg/mL)
SA	NA	NA	NA
TD-I	NA	750	NA
LOP	NA	NA	NA
CM-I	NA	NA	NA
PA	NA	NA	NA
EQ	NA	NA	NA
HD	NA	NA	NA
LOC	281	188	NA
LOCpT	375	281	NA
LOT-I	53	53	53
TD-II	BI	BI	BI
SV	NA	NA	NA
PS	NA	NA	NA
VC	NA	NA	NA
OB	NA	NA	NA
CM-II	NA	750	NA
TD-III	NA	750	NA
LOTC	94	141	141
LOT-II	188	188	281
LM	375	NA	NA
PM	NA	NA	NA
PR	NA	NA	NA
CaM	NA	NA	NA
AP-I	NA	NA	NA

Essential Oil	*C. albicans* ATCC 10231	*C. parapsilosis* ATCC 22019	*C. auris CDC B11903*
	$MBIC_{50}$ (µg/mL)	$MBIC_{50}$ (µg/mL)	$MBIC_{50}$ (µg/mL)
SF-I	NA	NA	NA
Vcu	NA	NA	NA
OC	NA	NA	NA
LACL	NA	750	NA
LACi	NA	NA	NA
PC	NA	NA	NA
HS	NA	NA	NA
MM-I	NA	NA	NA
MM-II	NA	NA	188
HC	NA	NA	NA
SO	NA	NA	NA
AP-II	NA	NA	NA
LAC	NA	NA	NA
PAu	NA	NA	NA
SF-II	NA	NA	NA
OA	NA	NA	NA
PS	NA	NA	NA
PD	NA	NA	NA
VCy	NA	NA	NA
AdP	NA	NA	NA
LC	NA	NA	NA
DC	NA	NA	NA
PP	BI	BI	BI
DF	NA	NA	NA
MR	NA	NA	NA
PAm	NA	NA	NA

BI: Biofilm increase; NA: Non-Active.

Table S5. Spearman´s correlation analysis between antifungal activities (MIC) against *Candida* strains vs. identified compounds in the Colombian EOs.

Compounds	Spearman´s correlation (r^2)		
	C. albicans ATCC 10231	*C. parapsilosis* ATCC 22019	*C. auris* CDC B11903
**Thymol**	**-0.6209 ******	**-0.6539 ******	**-0.5938 ******
**Thymyl methyl ether**	**-0.5640 ******	**-0.5505 ******	**-0.5207 *****
**Carvacrol**	**-0.5506 ******	**-0.4850 *****	**-0.5213 *****
**γ-Terpinene**	**-0.4249 ****	**-0.4418 ****	**-0.4769 *****
***p*-Cymene**	**-0.4206 ****	**-0.4316 ****	**-0.3504 ***
**Thymyl acetate**	**-0.2934 ***	**-0.3477 ****	-0.2634
***cis*-β-Ocimene**	**-0.2792 ***	-0.2164	-0.2533
**Humulene epoxide II**	**-0.2792 ***	-0.2651	-0.2758
**Terpinen-4-ol**	**-0.2792 ***	-0.2651	-0.2758
trans-β-Bergamotene	-0.2617	-0.2434	-0.2533
Carvacryl acetate	-0.2501	-0.2164	-0.2308
β-Myrcene	-0.2343	-0.0600	-0.2109
Nonane	-0.2327	-0.1893	-0.2139
Tricyclene	-0.2327	-0.1893	-0.2139
**α-Terpinene**	-0.2086	**-0.2814 ***	**-0.2815***
α-Selinene	-0.1724	-0.2027	-0.1178
Camphene	-0.1517	0.0570	0.1101
6-Hydroxycarvotanacetone	-0.1512	-0.1893	-0.2139
Aciphyllene	-0.1512	0.1298	0.1126
Geranial	-0.1512	-0.0866	-0.1914
Geraniol	-0.1512	-0.0866	-0.1914
Geranyl acetate	-0.1512	-0.0866	-0.1914
Methyl-eugenol	-0.1512	-0.0866	-0.1182
Neral	-0.1512	-0.0866	-0.1914
Nerol	-0.1512	-0.0866	-0.1914
Patchoulol	-0.1512	0.1298	0.1126
Piperitenone oxide	-0.1512	-0.1893	-0.2139
Pogostol	-0.1512	0.1298	0.1126
Seychellene	-0.1512	0.1298	0.1126
trans-Piperitone oxide	-0.1512	-0.1893	-0.2139
Verbenone	-0.1512	0.1298	0.1126
α-Bisabolol	-0.1512	0.1298	0.1126
α-Bulnesene	-0.1512	0.1298	0.1126
α-Guaiene	-0.1512	0.1298	0.1126
α-Patchoulene	-0.1512	0.1298	0.1126
β-Patchoulene	-0.1512	0.1298	0.1126
γ-Patchoulene	-0.1512	0.1298	0.1126
Aristolochene	-0.1105	-0.0306	0.0576
Germacra-4,5,10-trien-1-α-ol	-0.1105	-0.0255	0.0576

Compounds	Spearman´s correlation (r^2)		
	C. albicans ATCC 10231	*C. parapsilosis* ATCC 22019	*C. auris* CDC B11903
α-Humulene	-0.0978	-0.2724	-0.0539
Eugenol	-0.0866	-0.1804	-0.2201
Caryophyllene oxide	-0.0718	-0.1446	0.1599
β-Selinene	-0.0589	0.0522	0.0816
Dehydrofukinone	-0.0533	0.0446	0.1108
Valencene	-0.0533	0.0446	0.1108
α-Eudesmol	-0.0369	-0.2379	-0.1689
Carvone	-0.0369	0.0278	-0.2673
trans-Verbenol	-0.0369	0.1855	0.1608
Elemicin	-0.0369	0.0278	-0.0073
Menthone	-0.0296	-0.1956	-0.2358
Norneol	-0.0296	-0.1236	-0.2634
Piperitenone	-0.0296	0.0340	-0.2634
Terpinolene	-0.0296	0.0340	-0.1689
trans-β-Farnesene	-0.0296	0.0340	-0.2634
Premnaspirodiene	0.0007	-0.0540	0.1461
1,8-Cineole	0.0009	-0.0177	0.0801
Linalool	0.0107	-0.0084	-0.0240
Guaiol	0.0343	-0.0255	-0.0730
Pulegone	0.0403	-0.0785	-0.1216
α-Thujene	0.0403	0.0184	0.0685
β-Phellandrene	0.0478	0.0465	0.0760
Piperitone	0.0866	-0.0736	-0.1859
β-Pinene	0.0940	0.2020	0.1078
α-Phellandrene	0.0993	0.0125	0.1286
Iso-menthone	0.1047	-0.0866	-0.1182
Aromadendrene	0.1047	0.1298	0.1126
Camphor	0.1047	0.1298	0.1126
Carotol	0.1047	-0.0866	0.1126
Caryophylla-4(12),8(13)-dien-5-β-ol	0.1047	0.1298	0.1126
cis-3-Pinocamphone	0.1047	0.1298	-0.1182
cis-trans-Farnesol	0.1047	0.1298	0.1126
di-*epi*-1,10-Cubenol	0.1047	0.1298	0.1126
epi-α-Acoradiene	0.1047	0.1298	0.1126
Estragole	0.1047	0.1298	0.1126
Fenchone	0.1047	0.1298	0.1126
Ishwarol B	0.1047	0.1298	0.1126
Ishwarone	0.1047	0.1298	0.1126
Iso-spathulenol	0.1047	0.1298	0.1126
Ledol	0.1047	0.1298	0.1126
Myrtenol	0.1047	0.1298	0.1126
Palustrol	0.1047	0.1298	0.1126

Compounds	Spearman´s correlation (r^2)		
	C. albicans ATCC 10231	*C. parapsilosis* ATCC 22019	*C. auris* CDC B11903
p-Cumenol	0.1047	0.1298	-0.1182
Pinocarvone	0.1047	0.1298	-0.1182
p-Menth-3-en-8-ol	0.1047	0.1298	0.1126
p-Mentha-3,8-diene	0.1047	0.1298	0.1126
Safrole	0.1047	0.1298	-0.1182
trans-Pulegol	0.1047	0.1298	0.1126
trans-Sabinol	0.1047	0.1298	0.1126
trans-Thujanol	0.1047	0.1298	0.1126
trans-α-Bergamotene	0.1047	0.1298	0.1126
trans-β-Bisabolene	0.1047	-0.0866	0.1126
trans-β-Guaiene	0.1047	0.1298	0.1126
trans-β-Ocimene	0.1047	0.1298	0.1126
α-Cadinol	0.1047	0.1298	0.1126
α-Campho*lenal	0.1047	0.1298	-0.1182
α-Gurjunene	0.1047	0.1298	0.1126
β-Eudesmol	0.1047	0.1298	0.1126
γ-Cadinene	0.1047	0.1298	0.1126
γ-Elemene	0.1047	0.1298	0.1126
δ-Cadinol	0.1047	-0.0866	0.1126
δ-Elemene	0.1047	0.1298	0.1126
Eudesma-4(15),7-dien-1β-ol	0.1047	0.1298	0.1126
γ-Muurolene	0.1047	0.1298	0.1126
β-Burbonene	0.1151	-0.0968	-0.1326
Limonene	0.1267	0.1449	0.0052
Benzyl benzoate	0.1496	0.1855	0.1608
Elemol	0.1496	0.0340	-0.0007
epi-α-Cadinol	0.1496	0.1855	0.1608
Ishwarane	0.1496	0.1855	0.1608
Trans,trans-geranyl Linalool	0.1496	0.1855	0.1608
Trans-9-*epi*-Caryophyllene	0.1496	0.1855	-0.0007
Viridiflorine	0.1496	0.1855	0.1608
β-Cubebene	0.1496	-0.0379	0.1608
Δ^3-carene	0.1496	-0.0379	0.1608
Dillapiole	0.1496	0.0340	0.1608
Spathulenol	0.1496	0.1855	0.1608
Trans-β-caryophyllene	0.1512	-0.0627	0.2151
Selina-3,7(11)-diene	0.1851	0.2295	0.1990
Viridiflorol	0.1851	0.1020	0.1990
β-Elemene	0.2016	0.1383	0.1229
**α-Copaene**	0.2586	0.1803	**0.3187 ***
trans-Nerolidol	0.2699	0.1376	0.1811
**Germacrene B**	0.2699	0.2492	**0.2902 ***

Compounds	Spearman´s correlation (r^2)		
	C. albicans ATCC 10231	*C. parapsilosis* ATCC 22019	*C. auris* CDC B11903
Germacrene D	**0.2858 ***	0.1133	0.1301
Sabinene	**0.2946 ***	0.1948	0.1201
δ-Cadinene	**0.3031 ***	0.1580	0.3525 *
α-Pinene	**0.3375 ***	0.3658 **	0.4038 **
Bicyclogermacrene	**0.4142 ****	0.2826 *	0.2653

*: p ≤ 0.05; **: p ≤ 0.01; ***: p ≤ 0.001;****: p ≤ 0.0001

-1 **1**

Table S6. Spearman´s correlation analysis between antibiofilm (MBIC) activities against *Candida* strains vs. identified compounds in the Colombian EOs.

Compounds	Spearman´s correlation (r2)		
	C. albicans ATCC 10231	*C. parapsilosis* ATCC 22019	*C. auris* CDC B11903
**Carvacrol**	**-0.7350 ******	**-0.6477 ******	**-0.6066 ******
**Thymyl methyl ether**	**-0.8070 ******	**-0.5496 ******	**-0.5285 ******
**Thymol**	**-0.8318 ****	**-0.5985 ******	**-0.5030 *****
***trans*-β-Bergamotene**	**-0.3800 ****	**-0.3347 ***	**-0.4297 ****
***cis*-β-Ocimene**	**-0.3645 ****	**-0.3211 ***	**-0.4122 ****
**Iso-menthone**	0.0310	0.0478	**-0.3946 ****
**Humulene epoxide II**	**-0.3490 ***	**-0.3006 ***	**-0.3771 ****
**Terpinen-4-ol**	**-0.3490 ***	**-0.3006 ***	**-0.3771 ****
**γ-Terpinene**	**-0.6948 ******	**-0.4649 *****	**-0.3105 ***
**Thymyl acetate**	**-0.4920 *****	-0.1995	**-0.2880 ***
Menthone	0.0443	0.0683	-0.2752
Pulegone	0.0548	0.0845	-0.2118
**β-Myrcene**	**-0.3228 ***	-0.2109	-0.1942
***p*-Cymene**	**-0.4850 *****	**-0.3377 ***	-0.1859
Piperitone	0.0640	-0.0498	-0.1806
**α-Terpinene**	**-0.4282 ****	-0.2166	-0.1673
α-Humulene	-0.0810	0.0210	-0.1241
Caryophyllene oxide	-0.1675	-0.1681	-0.0930
**Carvacryl acetate**	**-0.3335 ***	**-0.3006 ***	0.0175
Nonane	0.0310	0.0478	0.0175
Tricyclene	0.0310	0.0478	0.0175
6-Hydroxycarvotanacetone	0.0310	0.0478	0.0175
Aciphyllene	0.0310	0.0478	0.0175
Geranial	0.0310	0.0478	0.0175
Geraniol	0.0310	0.0478	0.0175
Geranyl acetate	0.0310	0.0478	0.0175
Methyl-eugenol	0.0310	0.0478	0.0175

Compounds	Spearman´s correlation (r2)		
	C. albicans ATCC 10231	*C. parapsilosis* ATCC 22019	*C. auris* CDC B11903
Neral	0.0310	0.0478	0.0175
Nerol	0.0310	0.0478	0.0175
Patchoulol	0.0310	0.0478	0.0175
Piperitenone oxide	0.0310	0.0478	0.0175
Pogostol	0.0310	0.0478	0.0175
Seychellene	0.0310	0.0478	0.0175
trans-Piperitone oxide	0.0310	0.0478	0.0175
Verbenone	0.0310	0.0478	0.0175
α-Bisabolol	0.0310	0.0478	0.0175
α-Bulnesene	0.0310	0.0478	0.0175
α-Guaiene	0.0310	0.0478	0.0175
α-Patchoulene	0.0310	0.0478	0.0175
β-Patchoulene	0.0310	0.0478	0.0175
γ-Patchoulene	0.0310	0.0478	0.0175
Aromadendrene	0.0310	0.0478	0.0175
Camphor	0.0310	0.0478	0.0175
Carotol	0.0310	0.0478	0.0175
Caryophylla-4(12),8(13)-dien-5-β-ol	0.0310	0.0478	0.0175
cis-3-Pinocamphone	0.0310	0.0478	0.0175
cis-trans-Farnesol	0.0310	0.0478	0.0175
di-*epi*-1,10-Cubenol	0.0310	0.0478	0.0175
epi-α-Acoradiene	0.0310	0.0478	0.0175
Estragole	0.0310	0.0478	0.0175
Fenchone	0.0310	0.0478	0.0175
Ishwarol B	0.0310	0.0478	0.0175
Ishwarone	0.0310	0.0478	0.0175
Iso-spathulenol	0.0310	0.0478	0.0175
Ledol	0.0310	0.0478	0.0175
Myrtenol	0.0310	0.0478	0.0175
Palustrol	0.0310	0.0478	0.0175
p-Cumenol	0.0310	0.0478	0.0175
Pinocarvone	0.0310	0.0478	0.0175
p-Menth-3-en-8-ol	0.0310	0.0478	0.0175
p-Mentha-3,8-diene	0.0310	0.0478	0.0175
Safrole	0.0310	0.0478	0.0175
trans-Pulegol	0.0310	0.0478	0.0175
trans-Sabinol	0.0310	0.0478	0.0175
trans-Thujanol	0.0310	0.0478	0.0175
trans-α-Bergamotene	0.0310	0.0478	0.0175

Compounds	Spearman´s correlation (r2)		
	C. albicans ATCC 10231	*C. parapsilosis* ATCC 22019	*C. auris* CDC B11903
trans-β-Bisabolene	0.0310	0.0478	0.0175
trans-β-Guaiene	0.0310	0.0478	0.0175
trans-β-Ocimene	0.0310	0.0478	0.0175
α-Cadinol	0.0310	0.0478	0.0175
α-Campho*lenal	0.0310	0.0478	0.0175
α-Gurjunene	0.0310	0.0478	0.0175
β-Eudesmol	0.0310	0.0478	0.0175
γ-Cadinene	0.0310	0.0478	0.0175
γ-Elemene	0.0310	0.0478	0.0175
δ-Cadinol	0.0310	0.0478	0.0175
δ-Elemene	0.0310	0.0478	0.0175
Eugenol	0.0443	0.0683	0.0251
α-Eudesmol	-0.2043	-0.1706	0.0251
Carvone	0.0443	-0.1457	0.0251
trans-Verbenol	0.0443	0.0683	0.0251
Norneol	0.0443	-0.1373	0.0251
Piperitenone	0.0443	-0.1373	0.0251
Terpinolene	0.0443	0.0683	0.0251
trans-β-Farnesene	0.0443	-0.1373	0.0251
Benzyl benzoate	0.0443	0.0683	0.0251
Elemol	0.0443	0.0683	0.0251
epi-α-Cadinol	0.0443	0.0683	0.0251
Ishwarane	0.0443	0.0683	0.0251
trans,trans-Geranyl Linalool	0.0443	-0.1457	0.0251
trans-9-*epi*-Caryophyllene	0.0443	0.0683	0.0251
Viridiflorine	0.0443	0.0683	0.0251
β-Cubebene	0.0443	0.0683	0.0251
Δ^3-carene	0.0443	0.0683	0.0251
α-Selinene	0.0548	-0.0886	0.0310
α-Thujene	-0.1382	0.0845	0.0310
Selina-3,7(11)-diene	0.0548	-0.0955	0.0310
Viridiflorol	0.0548	-0.0955	0.0310
Camphene	0.0723	0.1114	0.0409
β-Burbonene	0.0723	-0.0364	0.0409
Germacrene D	0.2011	0.2368	0.0410
trans-β-caryophyllene	0.0727	0.1399	0.0418
β-Phellandrene	0.0800	0.1233	0.0452
trans-Nerolidol	0.0800	-0.0004	0.0452
Germacrene B	0.0800	0.1233	0.0452

**Compounds**	**Spearman´s correlation (r2)**		
	C. albicans **ATCC 10231**	*C. parapsilosis* **ATCC 22019**	*C. auris* **CDC B11903**
Linalool	0.0873	0.0307	0.0494
Sabinene	0.0873	0.1346	0.0494
Limonene	0.0871	-0.0107	0.0494
α-Copaene	0.1077	0.0702	0.0609
1,8-Cineole	-0.1142	-0.0526	0.0646
α-Phellandrene	-0.0046	0.0619	0.0646
δ-Cadinene	0.1142	0.1760	0.0646
**β-Pinene**	0.2221	**0.2915 ***	0.0886
**α-Pinene**	0.2426	**0.2791 ***	0.1095
**Bicyclogermacrene**	0.2633	**0.3209 ***	0.1130
β-Elemene	0.2553	0.2231	0.2122
β-Selinene	0.2327	-0.1027	0.2213
Premnaspirodiene	0.2439	-0.0269	0.2438
Dehydrofukinone	0.2434	-0.0630	0.2483
Valencene	0.2434	-0.0630	0.2483
Guaiol	0.2478	0.0699	0.2592
Aristolochene	0.2639	-0.0831	0.2782
Germacra-4,5,10-trien-1-α-ol	0.2639	-0.0831	0.2782
**Elemicin**	**0.2832 ***	0.2644	**0.3074 ***
**Dillapiole**	**0.2929 ***	0.2724	**0.3190 ***
**Spathulenol**	**0.2929 ***	0.2724	**0.3190 ***
**Eudesma-4(15),7-dien-1β-ol**	**0.3723 ****	**0.3279 ***	**0.4209 ****
**γ-Muurolene**	**0.3723 ****	**0.3279 ***	**0.4209 ****

*: p ≤ 0.05; **: p ≤ 0.01; ***: p ≤ 0.001; ****: p ≤ 0.0001;

-1 [colour scale] **1**
